# A whole-cell *Lactococcus garvieae* autovaccine protects Nile tilapia against infection

**DOI:** 10.1371/journal.pone.0230739

**Published:** 2020-03-26

**Authors:** Patricia Bwalya, Bernard M. Hang’ombe, Amr A. Gamil, Hetron M. Munang'andu, Øystein Evensen, Stephen Mutoloki

**Affiliations:** 1 Faculty of Veterinary Medicine, Norwegian University of Life Sciences, Oslo, Norway; 2 Samora Machel School of Veterinary Medicine, University of Zambia, Lusaka, Zambia; 3 Department of Veterinary and Livestock Services, Ministry of Fisheries and Livestock, Lusaka, Zambia; Friedrich-Loeffler-Institut Bundesforschungsinstitut fur Tiergesundheit, GERMANY

## Abstract

The autovaccine was produced in-house using a bacterial isolate from a diseased fish from the target farm. Three groups of 150 fish each were injected with either 1) an oil-adjuvanted, inactivated whole cell autovaccine, 2) adjuvant only or 3) PBS (negative control). Approximately 660 degree days post vaccination, the fish were challenged with 9x10^5^ cfu bacteria/fish by intraperitoneal injection and monitored for a further 28 days. Protection against infections was measured by lack of/reduced bacterial loads both by bacterial re-isolation and immunohistochemistry as well as absence of clinical signs/pathology. Significantly less *L*. *garvieae* (p<0.03) was re-isolated from either the adjuvant only or control groups compared to the vaccinated group. Furthermore, a significantly high amount (p<0.001) of anti-*L*. *garvieae* specific antibodies were observed in the vaccinated group compared to the adjuvant only or control groups at time of challenge. This coincided with protection against infection measured by absence/reduced *L*. *garvieae* re-isolation from internal organs, reduced clinical signs and lack of pathology in this group. In the adjuvant only and control groups, bacteria were re-isolated from the kidney, liver, spleen, brain and eyes during the first 14 days. The findings suggest that oil-based vaccines can protect tilapia against *L*. *garvieae* infection through an antibody mediated response.

## Introduction

Tilapia farming in Zambia is a relatively young but rapidly growing industry. Although commercialization started as far back as the 1990s, the surge in production was not until 2010 that cage-based commercial farming intensified on Lake Kariba [1]. Zambia’s annual aquaculture production presently stands around 30,000 metric tons [2].

As with intensive fish farming elsewhere that is affected by fish diseases [3], the increase in the fish production on Lake Kariba soon faced the same problem. Lactococcosis outbreaks caused by *Lactococcus garvieae (L*. *garvieae)* have been experienced since early this decade [4].

*Lactococcus garvieae* is a facultatively anaerobic, non-motile, non-spore forming, Gram-positive, ovoid cocci bacteria belonging to the Phylum *Firmicutes*, Family *Streptococcaceae*, Order *Lactobacilles* and genus Lactococcus. It is well-known for infecting and causing disease in rainbow trout [5, 6] and yellowtail (*Seriola quinqueradiata*) [7]. Clinical signs include exophthalmia, conjunctivitis, melanosis, erratic swimming, anorexia, internal hemorrhage and congestion of blood vessels, peritonitis, meningoencephalitis and septicaemia [8–12].

In tilapia, *L*. *garvieae* infections are a cause of an emerging disease that became of major importance during the last decade [13, 14]. Infections are most severe when water temperatures are above 20°C [14, 15]. Economic losses occur as a result of mortalities (high or low), downgrading of carcasses due to unsightly skin lesions and reduced growth rate [8, 13, 14]. No protective commercial vaccines for tilapia are available on the market at the moment.

The objective of this study was to develop a whole bacterial cell autogenous oil-based vaccine for the protection of tilapia against *L*. *garvieae* infections. Autogenous vaccines are custom-made that are produced on a small to medium scale; and are based on pathogens isolated from a farm on which they are to be used. They have the advantage of being less amenable to rigorous regulations applicable to commercial vaccines [16] and allow for more rapid availability without complete and comprehensive characterization in the face of an outbreak [17].

To evaluate the effect of the vaccine, protection against infection was done by bacterial re-isolation and immunohistochemistry supported by clinical signs/ pathology. Infection is a pre-requisite of disease and therefore a good proxy for total protection.

## Materials and methods

This study was undertaken in strict accordance with the recommendations in the Guide for the Care and Use of Laboratory Animals of the National Health Research Ethics Committee of Zambia. The protocol was approved by the Excellence in Research Ethics and Science (ERES) Converge, a private Research Ethics Board (Protocol Number: 2016/JUNE/028). All efforts were made to minimize suffering and stress of the fish, both during handling and sampling. As infection was one of the humane endpoints, subjects were withdrawn from the experiment (euthanized and sampled) before clinical signs appeared. Where signs of disease or abnormal behaviour (lethargy, disorientation, aberrant swimming etc) were observed, the fish were euthanised by stunning with a blow to the head followed by dislocation of the cervical vertebra.

### Fish

A total of 460 healthy Nile tilapia (*Oreochromis niloticus*) with mean weight of 41.5 ± 16.5g were purchased from Palabana fisheries, a commercial fish farm located in Chirundu district, South-East of Zambia. The fish farm had no previous history of disease outbreaks and the subjects were transported by road in oxygenated bags to the University of Zambia, School of Veterinary Medicine wet-lab. The fish were kept in 500 L tanks supplied with flow-through de-chlorinated water and aerated using stone bubblers. They were allowed to acclimatize for 10 days prior to commencement of the experiment. The fish were fed daily on commercial pellets at a rate of 3% body weight. Daily water temperature averaged 20 ±2°C, mean daily dissolved oxygen was 7.9 ± 2 mg/L and pH was 7 ± 0.2.

### Antigens and vaccine formulation

*Lactococcus garvieae* previously isolated from a diseased fish at a farm on Lake Kariba with a partial 16S RNA sequence (Genbank accession number MK346137) and stored in Tryptic Soy Broth (TSB, HiMedia, India) and 20% glycerol was used. The bacteria was propagated in TSB and incubated at 37°C on a shaker at 180 rpm for 72 hours. Thereafter, it was centrifuged at 800 x g for 19 min at room temperature to pellet the cells. The bacteria was then inactivated with 4% formalin for 72h followed by dialysis using PBS for 48h. The inactivation process was confirmed by inoculation of the antigen on nutrient agar followed by incubation at 37°C for 72h to demonstrate the absence of bacterial growth. The vaccine was formulated using 10^9^ cfu/mL as a water-in-oil emulsion using the ISA 763 VG adjuvant (Seppic, France) according to manufacturer’s guidelines. The adjuvant only group was prepared in the same way but without bacteria. The preparations were then stored at 4°C until used. The vaccine and adjuvant only were determined to be sterile by lack of bacterial growth on nutrient and Sheep's Blood Agar after 72h incubation at 37°C.

### Vaccination of fish

Prior to the start of the study, 10 fish were sacrificed, sampled and tested for the presence or absence of bacterial infections by bacteria re-isolation (described below). Furthermore, ELISA was also done on serum from these fish to confirm that the fish had not recently been in contact with *L*. *garvieae*.

**Fig 1:**
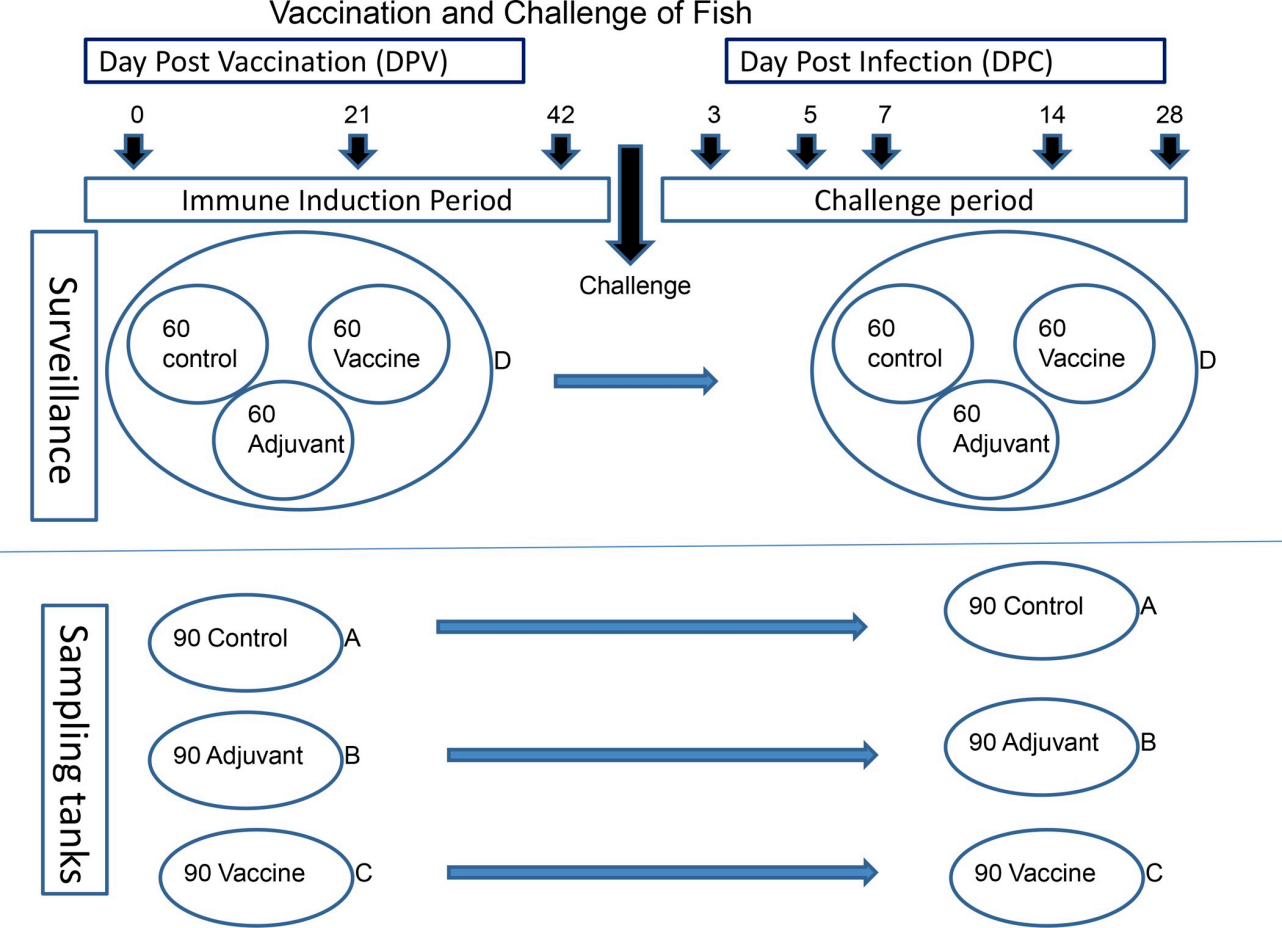
Schematic diagram showing the experimental design of the present study. Different groups of fish were injected intraperitoneally with the indicated preparations and allowed a period of 42 days to mount an immune response. Thereafter, the fish were challenged with 9 x 10^5^ cfu of bacteria per fish. Key: Cont = control (PBS) group; Vac = vaccinated group; Adj = adjuvant only group; DPV = days post vaccination: DPC = Days post challenge.

The fish (n = 450) were divided into in 3 groups (Control, Adjuvant and Vaccine) by dip-netting and sequential allocation. The control fish were injected with PBS: Adjuvant group were injected with adjuvant only and the Vaccine group with *L*. *garvieae* vaccine. The total number of fish per group was 150 individuals. Each group was further split into two replicates, one for observation (surveillance) and the other for sampling (Fig 1). For sampling, each group was placed in a separate tank (A-C), each containing 90 individuals. The rest of the fish were pooled together in tank D (surveillance), containing 60 control, 60 vaccinated and 60 adjuvant-only groups all mixed together. The fish in tank D were marked by clipping of the dorsal fin, caudal fin or left unclipped to differentiate between groups. All fish were injected intraperitoneally with 0.1ml of vaccine, adjuvant-only or PBS.

### Challenge experiment

Following vaccination, all fish in the four tanks (A, B, C and D) were allowed a period of 6 weeks for immune induction (Fig 1). On day 43 (approximately 660 degree days) post vaccination (dpv), the fish were challenged by intraperitoneal injection of 0.1 ml of *L*. *garvieae* suspension (9.6 x10^5^ cfu bacteria/fish). Monitoring was done for 28 dpc during which clinical signs were recorded and sampling for bacterial re-isolation was done.

### Sample collection

At each sampling time point on days 21 and 42 post vaccination; and 3, 5, 7, 14, and 28 post challenge, 10 fish per group from tanks A, B and C were sampled by dip-netting and anaesthetized by using Benzocain (Sigma-Aldrich, Germany) at a dosage of 5 ml/L. Blood was first collected from the caudal vein into non-heparinised tubes. Thereafter, bacteriological samples were collected by aseptically inserting a sterile loop into each of the liver, kidney, spleen, eyes and brain and then streaking directly on nutrient agar plates (HiMedia, India). The plates were then incubated aerobically at 24°C for 48 hours. Parallel samples were also collected in 10% phosphate-buffered formalin.

### Clinical signs of disease post-challenge

Fish were observed twice daily for clinical signs. Furthermore, the fish were inspected during sampling for any clinical signs. Surveillance for clinical signs or mortalities was done primarily in tank D although attention to other tanks was also paid.

### Production of hyperimmune serum

Production of hyperimmune serum against *L*. *garvieae* was outsourced to the Section for Experimental Biomedicine at the Norwegian University of Life Sciences in Oslo, Norway. The animal facility is licensed by the Norwegian Food Safety Authority (https://www.mattilsynet.no/language/english/) and accredited by the Association for Assessment and Accreditation of Laboratory Animal Care (https://www.aaalac.org/). The animal experiment was approved by the unit’s animal ethics committee (Institutional Animal Care and Use Committee/IACUC) and the Food Safety Authority (application ID: FOTS 13566) and executed in compliance with the local and national regulations associated with laboratory animal experiments. The rodent and rabbit section of the facility is a Specific Pathogen Free (SPF) unit and follows a health monitoring program recommended by Federation of European Laboratory Animal Science Associations/FELASA (http://www.felasa.eu/). The care of the animals was carried out by two veterinary nurses with FELASA B certification and the study was performed by a veterinarian with FELASA C certification.

*Lactococcus garvieae* previously isolated from a diseased fish at one farm on Lake Kariba with partial 16S RNA sequence (Genbank accession number MK346137) was used. The bacteria was propagated in 300 ml of TSB in a fermenter at 37°C until an optical density (OD) of 0.6 at 595 nm was reached. Bacterial cells were collected by centrifugation at 800 x g for 19 min at room temperature and then inactivated with 4% formalin for 72h. Bacterial cells were collected again by centrifugation and washed in PBS three times. Finally, the cells were resuspended in PBS to OD_595_ 0.6.

A rabbit reared according to standard procedures was used to produce hyperimmune serum. The rabbit was first allowed to acclimatize for 2 weeks prior to the first (primary) injection of *L*. *garvieae* emulsified in Freund’s complete adjuvant. The rabbit was injected with 10^8^ cfu bacteria in 0.5 ml. All booster vaccines were formulated in Freund’s incomplete adjuvant and were administered on days 14 (1^st^ booster), 21 (second booster) and 28 (third booster). Blood samples were collected before the first injection and on day 28 before the third booster. The terminal bleeding was done on day 42.

### Specificity and reactivity of the hyperimmune serum against other bacteria

To measure the titres of immunoglobulins rabbit serum prior- and post-harvest, ELISA plates (NUNC, Maxisorp) were first coated with 100 ul per well of *L*. *garvieae* (0.6 OD_595_). Prior to this, the bacteria was diluted in 0.1 M coating buffer (0.1 M Carbonate buffer, pH 9.6) in 5mL suspension and then homogenized in a mixer mill (Anders Pihl As) at 30s per min. Plates coated with two of these bacterial dilutions (1:100 and 1:1000) were incubated overnight at 4°C. Following incubation, the plates were washed three times with 250ul washing buffer (1 L PBS plus 0.5 mls Tween 20 (PBST)) per well to remove unbound antigen. 250ul of blocking buffer (5% fat-free dry milk in PBS) was added to each well and plates incubated at room temperature for 2 hours after which the they were washed as described above. 100ul of rabbit *L*. *garvieae* serially diluted antiserum (1:100, 1:1000, 1:10000, and 1:20000) was added and plates incubated at room temperature for 1 hour. Plates were then washed and 100ul of goat anti-rabbit polyclonal antibody conjugated with Horse Radish Peroxidase (HRP) (GE Healthcare, UK) diluted to 1:1000 in 1% fat-free dry milk was added to each well and incubated at room temperature for 1 hour. After washing, 100ul of O-Phenylenediamine Dihydrochloride (OPD) substrate containing H_2_0_2_ (DAKO, Denmark) prepared according to manufacturer’s recommendations was added. The plates were incubated at room temperature in the dark and the reaction was stopped by adding 50 ul of (55mls of 1M H_2_SO_4_ in 945mls water) after 10 min. A spectrophotometer (Tecan GENios) was used to read the absorbance at 492nm.

To test the specificity of the hyperimmune serum, Elisa was used. First, the serum was adsorbed by dilution 1:1000 in PBS containing 0.57 OD_595_ of *L*. *garvieae* followed by adsorbed for 2 hours at 37°C. The mixture was centrifuged at 8000 x g for 5 min to pellet bacterial cells and recover the serum. The serum was then used in an Elisa (as described above) as a primary antibody. Un-adsorbed antibodies were also used in parallel as positive controls.

For non-reactivity of the hyperimmune serum against other bacteria, *Streptococcus garvieae*, *Yersinia ruckeri*, *Aeromonas hydrophila* and *L*. *garvieae* were tested by Elisa. The different bacteria were coated on Elisa plates and the procedure performed as described above.

### Histopathology and immunohistochemistry

Formalin fixed tissues were used for histopathology and were processed according to standard procedures for haematoxylin and eosin staining.

For immunohistochemistry, paraffin-embedded sections were cut into ultra-thin slices of 3–4 μm onto poly-L-lysine coated glass slides. Dewaxing was done by incubating the slides at 58°C for 25 min followed by two incubation steps in xylene. The sections were rehydrated by graded alcohol incubations of 5 min each.

A ring was then drawn around the sections using a PAP-pen to prevent the solutions from flowing off the specimens. For antigen retrieval, the tissues were pretreated with 10 nmol of citrate buffer, pH 6.0 containing 10% of trypsin at 37°C for 10 min. All incubations were performed in humidified chambers at room temperature and about 150 μL of different reagents were added to each specimen. After pre-treatment, the slides were washed in ice-cold PBS twice for 10 min. 5% (w/v) bovine serum albumin (BSA) in TBST for 2 hours was used to block non-specific binding. Thereafter, the slides were incubated with rabbit anti-*L*. *garvieae* serum (diluted 1: 500 in 2.5% BSA in TBST, see below). Non-immune serum was also included as a negative control. This was followed by incubation overnight at 4°C. Washing was done twice in TBST for 10 min and unless otherwise stated, all washing steps were done this way. Next, biotinylated goat anti-rabbit immunoglobulin (1:300 in 2.5% BSA in TBST) was added and incubated for 30 min. After washing, the sections were covered with Streptavidin-Alkaline Phosphatase Conjugate (Roche, Germany) and incubated for 30 min. After another wash, the sections were treated with Diaminobenzidine (DAB) (Sigma, USA) prepared as per manufacturers containing 3% H_2_O_2_ for 5 min. After this, washing was done in running water for 5 min, followed by counter-staining with Mayer’s modified hematoxylin for 30 seconds. The slides were then washed in running water for 5 min and mounted using Aquamount. For negative controls, rabbit anti-*L*. *garvieae* antibody was replaced with non-immune rabbit serum.

### Enzyme-linked immunosorbent assay (ELISA)

Blood samples collected from fish as described in the sample collection section were used. The blood was allowed to clot at 4°C. The serum was separated by centrifugation at 1204 x g, 4°C for 10 min and then stored in separate tubes at -20°C until required.

Preparation of the bacteria and coating of plates was done as described in the section of the production of hyperimmune serum. ELISA plates (NUNC, Maxisorp) were coated with 100 μL of homogenized *L*. *garvieae* (0.6 OD_595_) in PBS and incubated overnight at 4°C. Unbound antigens were removed by washing with 250 μL per well with PBS and 0.5% Tween20 (PBST), 3 times. Thereafter, 250 μL per well of blocking buffer (5% (w/v) Bovine Serum Albumin (BSA in PBST) was added and incubated at room temperature for 2 hours. The plates were washed 3 times as described above. Diluted serum (from the experimental fish) in PBS (1/40 or 1/80) was added (100 μL per well) to the ELISA plates and incubated overnight at 4°C. The plates were then washed followed by incubation with 100 μL per well of monoclonal anti-tilapia IgM antibody ((1:30 dilution) Aquatic Diagnostics, Britain) and incubation for 60 min at room temperature. After another washing step, 100 μL per well of rabbit anti-mouse IgG-HRP diluted 1/1000 in PBST was added and incubated at room temperature for an hour. Following washing, 100 μL per well of O-Phenylenediamine Dihydrochloride (OPD) substrate containing H_2_O_2_ (DAKO, Denmark) prepared as per manufacturers recommendations was added. The plates were incubated for 10 min at room temperature after which the reaction was stopped by adding 50 μL per well of 2M Sulphuric acid. Reading of results was done using a spectrophotometer (Tecan GENios) at 492 nm.

### Statistical analysis

Fischer’s exact and Chi square tests were used to determine independence or association between the treatment groups and outcomes of infection measured either by bacterial re-isolation or immunohistochemistry with the help of the JMP program (SAS Institute, USA) or Graphpad Prism 5.0 (www.graphpad.com). Regression analysis was used to examine statistical significance between groups in the antibody responses. All statistical tests were done with 95% confidence level.

## Results

### Clinical signs and autopsy findings

No *L*. *garvieae* was detected in the 10 fish that were sacrificed and tested before the start of the experiment.

In experimental groups, clinical signs were observed mostly in the control (PBS) and adjuvant only groups (Table 1). The most common sign was ocular opacity, uni- or bilateral with or without exophthalmia. The frequency was highest in the control followed by the adjuvant only group. In the former, fish with clinical signs were first observed at 3 days post challenge (dpc) and culminated on 5 dpc. In the adjuvant group, the first onset of clinical signs was on 5 dpc followed by 7 dpc (Table 1). In the vaccinated group, only two fish showed clinical signs, one at 3 dpc, likely due to physical injury unrelated to challenge and another one at 14 dpc, this time with corneal opacity (Table 1). No mortalities were observed in any of the groups.

**Table 1:** Frequency of clinical signs observed in the different treatment groups in the present study. DPC = days post challenge.

DPC	Clinical finding	Treatment group (n = 10/time point/group)		
		Vaccination	Adjuvant	Control
**3**	Body wound /skin ulcer	0.1	0	0
	Bilateral ocular opacity plus exophthalmia	0	0	0.1
	Unilateral ocular opacity plus exophthalmia	0	0	0.1
**5**	Bilateral ocular opacity plus exophthalmia	0	0	0.1
	Bilateral ocular opacity	0	0.1	0
	Unilateral ocular opacity plus exophthalmia	0	0.2	0.3
**7**	Unilateral ocular opacity plus exophthalmia	0	0.2	0
**14**	Unilateral ocular opacity	0.1	0	0
	Bilateral ocular opacity plus exophthalmia	0	0.1	0
**28**	Swimming upside down plus wound below mouth	0	0.1	0

During autopsy, changes were observed in the control and adjuvant groups only (Table 2). As with clinical signs, lesions were first observed in the control groups, before onset in the adjuvant only group. No changes were observed in the vaccinated group at any of the sampling time points.

**Table 2:** Frequency of changes in fish observed during autopsy. DPC = days post challenge.

DPC	Autopsy finding	Treatment group (n = 10)		
		Vaccination	Adjuvant	Control
**3**	Pale liver plus enlarged spleen	0	0	0.3
	Pale liver	0	0	0.1
**5**	Enlarged spleen plus distended gall bladder	0	0.1	0
**14**	Pale liver plus enlarged spleen	0	0.2	0

### Bacterial re-isolation from tissues of fish from different treatment groups

Assessment of fish infected with *L*. *garvieae* was done by bacterial re-isolation from the kidney, spleen, liver, brain and eyes (n = 10) in each group per time point post challenge. Where *L*. *garvieae* was re-isolated from at least one organ, the fish was considered infected. In general, the number of infected fish was relatively low, with overall 20% of the control and 28% of the adjuvant only groups against 6% of the vaccinated group (Table 3). There was, however, a significant difference (p<0.02) in the number of fish infected by the bacteria between groups. Significantly less bacteria (p<0.03) was re-isolated from the vaccinated group compared to either the adjuvant only or the control groups. The results also showed that control fish were infected first (3 dpc) followed by the adjuvant only and vaccinated groups from which bacteria was re-isolated at 5 dpi. No *L*. *garvieae* was re-isolated from any fish from 14 to 28 dpc.

**Table 3:** Percentage fish infected with L. garvieae in different groups following challenge. Infection was assessed by bacterial re-isolation from internal organs of 10 fish per group at each time point.

Days post infection	Vaccinated	Adjuvant only	Control
3	0	30%	70%
5	30%	50%	30%
7	0	60%	0
14	0	0	0
28	0	0	0
Overall	6%	28%	20%

### Distribution of *L*. *garvieae* in different organs

In the vaccinated group, *L*. *garvieae* was only re-isolated from one organ (spleen) and this was on 5 dpc. In contrast, the bacteria was isolated from all organs in both the control and adjuvant groups. Furthermore, the trends of bacterial re-isolation from different organs from these groups suggested that the liver, kidney and spleen were infected first, followed by the eyes and finally the brain (Table 4). The control groups had the highest number of *L*. *garvieae* isolation on day 3, with less re-isolations at 5 dpc.

**Table 4:** Summary of the frequencies of *L*. *garvieae* re-isolation from different tissues. A sterile inoculation loop was pierced into each of the indicated organs during sampling and inoculated on Nutrient Agar (HiMedia, India) plates which, were then incubated at 24°C for 48 hours. No bacteria were re-isolated from any group on days 14 and 28 post challenge. n = 10.

Tissue	Treatment	Day post challenge		
		3	5	7
	Vaccinated	0	0	0
Kidney	Adjuvant	0.1	0.3	0
	Control	0.5	0	0
	Vaccinated	0	0	0
Liver	Adjuvant	0	0.2	0
	Control	0.3	0	0
	Vaccinated	0	0.3	0
Spleen	Adjuvant	0.2	0.4	0.1
	Control	0.5	0.2	0
	Vaccinated	0	0	0
Eyes	Adjuvant	0.2	0.4	0.2
	Control	0.3	0.3	0
	Vaccinated	0	0	0
Brain	Adjuvant	0	0	0.1
	Control	0.3	0.1	0

A Kruskal-Wallis test of these observations shows that there was a significant difference (p<0.03) between treatment groups with the vaccinated fish being lower. This was further analysed by a multiple logistic regression where the dependent variable was ‘positive bacterial identification (>0)’ and independent variables are treatment (control, adjuvant, or vaccinated), organs and days post challenge. The overall model was statistically significant (p = 0.000), chi2 = 30.7, with vaccination significantly lowering (p = 0.013) the odds ratio of positive bacterial growth from the defined organs (20-fold less likely than adjuvant and 15-fold compared to controls). Bacterial re-isolation also decreased with increasing days post challenge (p<0.01).

### *Lactococcus garvieae* hyperimmune serum

Rabbit hyperimmune serum was harvested on day 42, after *L*. *garvieae*-specific immunoglobulins reached an OD_595_ value of 1.55 against background pre-immunization OD of 0.4. The best bacterial concentration for coating the plates was 1/100 while the best antiserum working concentration was 1:1000. A lower concentration resulted in suppression of the assay while dilutions above 1:1000 resulted in weak signals.

No reactivity was observed when the hyperimmune serum was first adsorbed with *L*. *garvieae* (results not shown). Similarly, the serum did not cross-react with any of the bacteria (*S*. *agalactiae*, *A*. *hydrophila or Y*. *ruckeri*) tested.

### Immunohistochemistry

**Fig 2:**
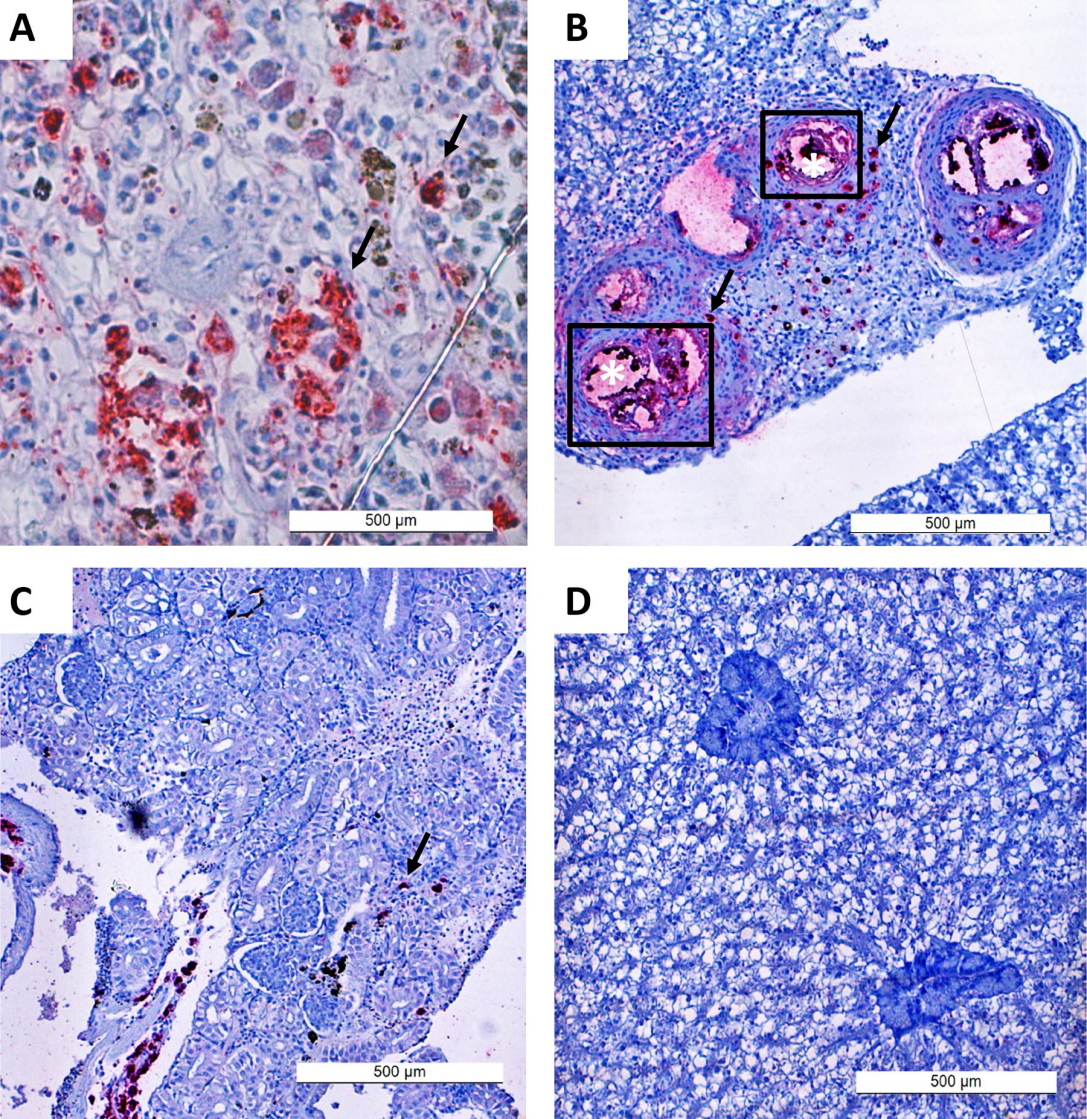
Immunohistochemistry staining of *L*. *garvieae* in different tissues of Nile tilapia following intraperitoneal injection. Bacterial antigens were observed as red stains (arrows) in the spleen (A), hepato-pancreas (liver) (B), the and kidney (C). In the liver (B), the bacteria is concentrated in and around blood vessels (*) which are likely portals of entry. Image (D) is a negative control (Liver), showing no positive stain.

Immunohistochemistry was done as an additional method to demonstrate the presence of *L*. *garvieae* in different tissues in the present study, immunohistochemistry was utilized. By this method, *L*. *garvieae* antigens were observed firstly around blood vessels and surrounding areas in tissues examined (hepato-pancreas (liver), kidney, spleen, brain and eyes, and also in interstitial areas (Fig 2). The most optimal titration of rabbit hyperimmune serum for use in immunohistochemistry assays was 1:500.

A significantly lower number of fish (p<0.01) with *L*. *garvieae* antigens was observed in the vaccinated group compared to the adjuvant only or controls. On the other hand, no difference (p = 0.05) in the number of fish with antigens was observed between the control and the adjuvant only groups.

**Fig 3:**
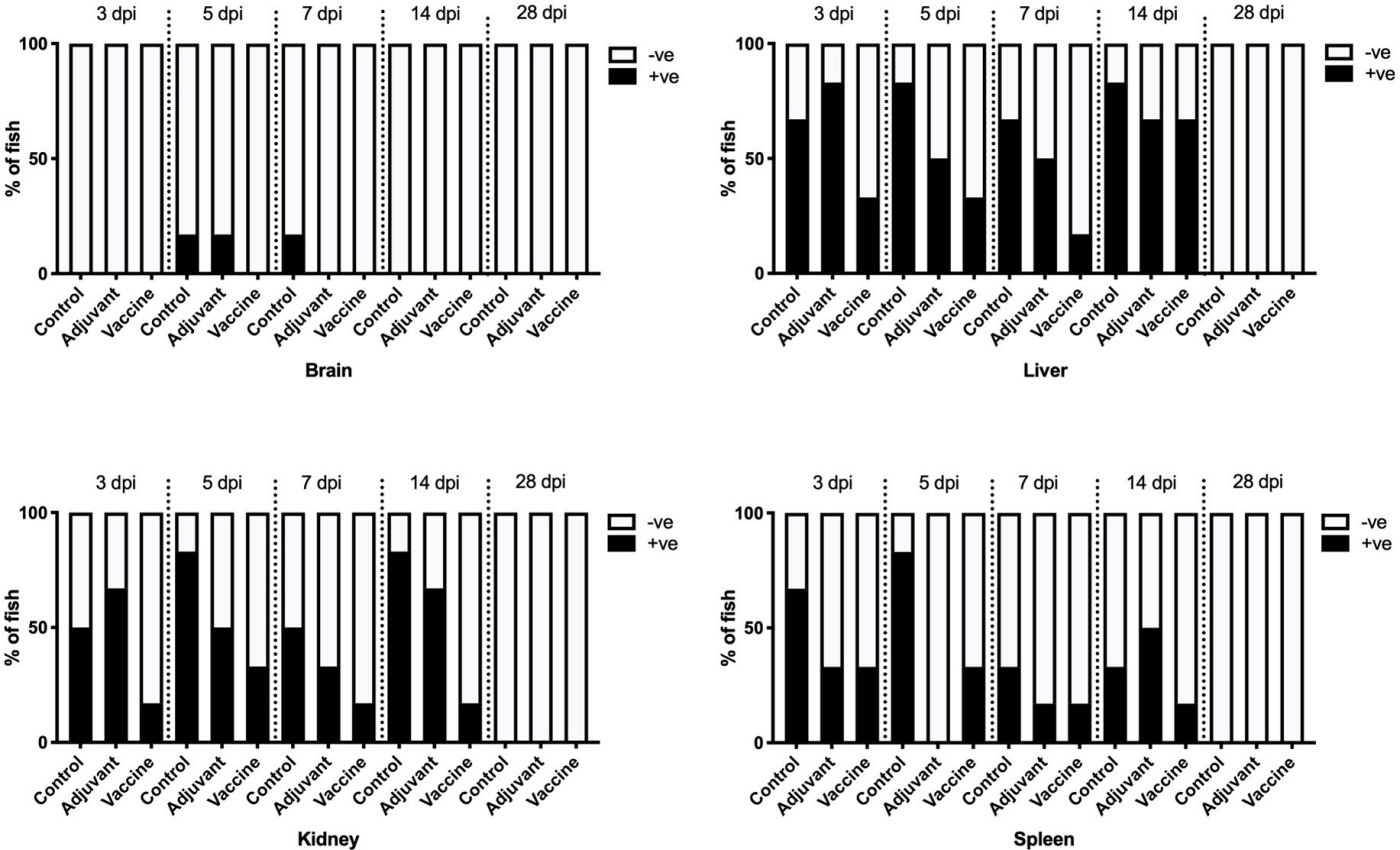
Percentage of fish organs positive for *L*. *garvieae* per group and organs (brain, liver, kidney and spleen) 3–28 days post challenge. At each sampling point, n = 10.

Unlike bacterial re-isolation, *L*. *garvieae* was demonstrated in all groups in the liver, kidneys and spleen starting with 3, 5, 7 and 14 dpc. No antigens were observed in any of the groups at 28 dpc. In the liver, at least half of the fish had antigens in the control and adjuvant only groups while fewer fish were positive in vaccinated group (Fig 3). A similar trend was observed in the kidneys and spleen for all groups. *L*. *garvieae* antigens was consistently with lower prevalence in vaccinated groups in all organs and over the entire course of the challenge period (Fig 3). Relative to the distribution of bacteria in different organs, the brain had the least, with few fish showing positive reaction at 5 dpi and 7dpi in controls and adjuvant groups, as well as at 14 dpi in the latter (Fig 3).

### Enzyme linked immunosorbent assay (ELISA)

For the assessment of fish responses to vaccination, fish sera diluted at 1/40 or 1/80 yielded similar results in this study and so for clarity, only results of 1/40 are presented. Fish were negative for *L*. *garvieae* specific antibodies prior to vaccination.

**Fig 4:**
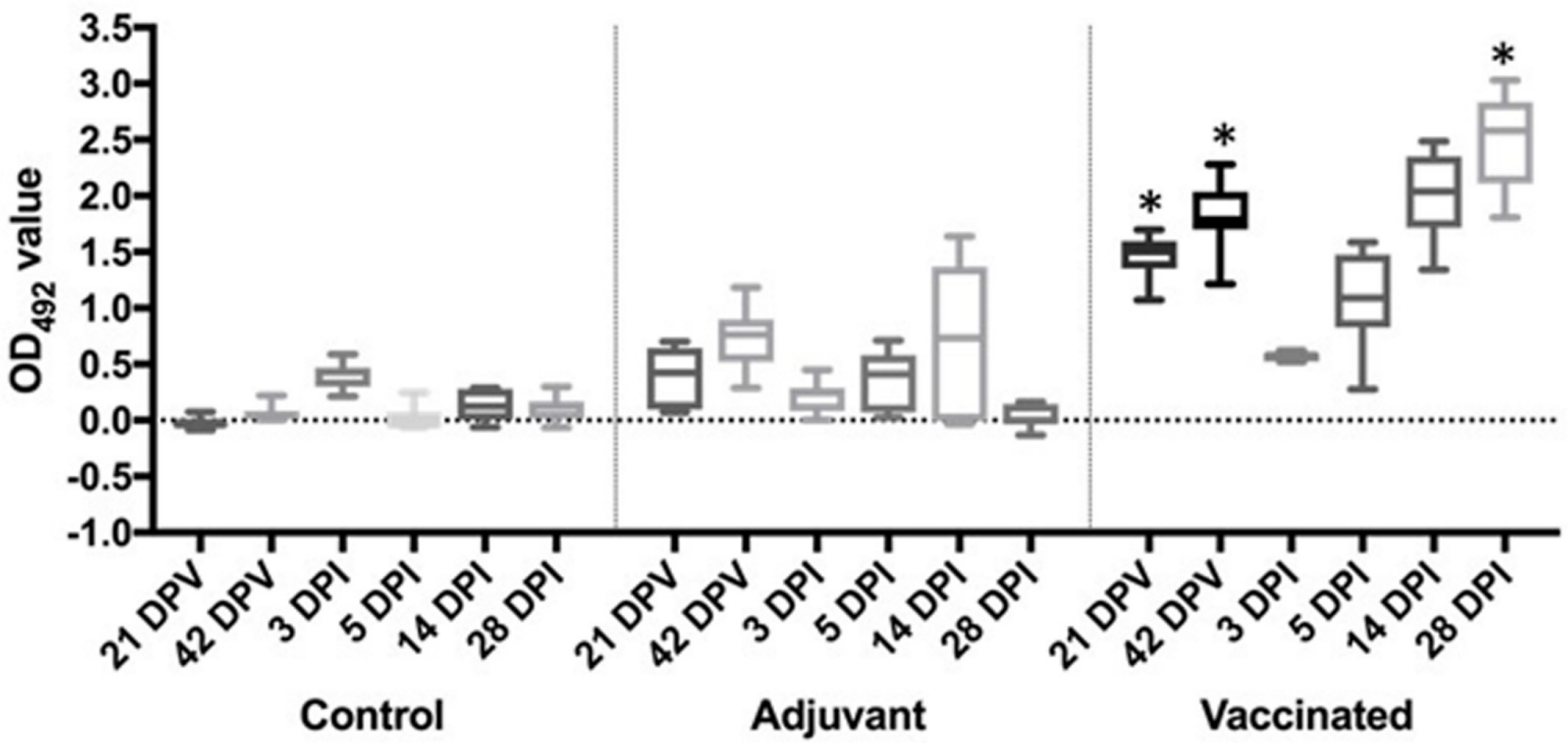
Antibody levels measured by ELISA shown as box-plots over time post vaccination (dpv) and days post challenge (dpc), expressed as OD_492_. Variations between groups were analysed by Regression analysis with 95% confidence. Note a non-specific increase in the adjuvant group and the drop in antibody levels by 3 dpi in vaccinated fish (and in adjuvant group). Key: *significantly different (at least p<0.05).

Following vaccination however, anti-*L*. *garvieae* specific antibody titres increased significantly (p<0.001) compared to non- vaccinated and adjuvant only groups at both days 21 and 42 post vaccination (Fig 4).

At 3 dpi, a marked drop in antibody levels was observed in the vaccinated group but this was followed by a steady increase from 5dpc onwards until the end of the study. In the control group, anti-*L*. *garvieae* remained at background levels throughout the priming period of 42 days. 3 days after challenge however, a slight increase was observed followed by a rapid return to background levels from 5 dpc (Fig 4). The response of fish in the adjuvant group was in-between, with a slight pre-challenge increase followed by a drop at 3 dpi and a slight but non-significant increase in antibody titres following challenge.

## Discussion

*Lactococcus garvieae* infections in farmed tilapia became a problem in the industry during the last decade [8, 18, 19]. There are very few reports on studies of efficacious vaccines against this bacterial infection in tilapia besides Tsai and co-workers [19] who reported full protection with inactivated whole bacteria combined with recombinant GADPH. The primary objective of the present study was to develop a protective oil-based, autogenous vaccine for the protection of tilapia on Lake Kariba. Our findings suggest that a whole bacterial cell autovaccine can protects Nile tilapia against *L*. *garvieae* infection. The vaccinated group in the present study was significantly more protected than the adjuvant only (p = 0.004) or the control (p = 0.03) groups. These findings support the reports of Tsai and others [19] that oil-adjuvanted vaccines can induce protection of tilapia against infection with *L*. *garvieae*. Our findings further suggest that the mechanism by which this is achieved is likely via an antibody mediated response. No clinical signs or post-mortem changes were observed in the vaccinated group nor was *L*. *garvieae* re-isolated from any of the tissues (except spleen of one fish) at any time point following challenge (Table 4). In contrast, clinical signs and post-mortem changes were observed in the adjuvant only and unvaccinated control groups (Tables 1 and 2) during the first 14 days following challenge. Furthermore, significantly more *L*. *garvieae* was re-isolated from the control (p = 0.04) and adjuvant only (p = 0.004) groups in this study during the first 7 days. No bacteria was re-isolated from any fish in any group from 14 dpc. The reason for this is yet to be investigated but *L*. *garvieae* under experimental conditions has been shown to induce acute infections [20], typically within 10 days post infection. Fish that do not succumb within this time recover from the infection and it is not unlikely that they clear the bacteria with time.

The low prevalence of infection in the present study contrast reports of others [19, 20] where significant mortalities were observed following challenge of tilapia with *L*. *garvieae*. A major difference between this and the two studies by Tsai and colleagues was the dosage used (10^5^ CFU in the present study versus 10^8^ CFU in the other studies). 9.6 x 10^5^ CFU/fish was used in the present study as determined by LD50 in an earlier study [21] according to standard procedures of determining infectious dosages for bacteria [22]. It is not explained how the dosage of 10^8^ was arrived at in the other two studies [19, 20]. The difficulty associated with challenge models is that huge investments both in terms of time and resources are required to optimize them through pathogen characterization, host susceptibility, optimal environmental conditions etc. [23]. Hence alternative, equally effective methods that can rapidly provide estimates for protection and are in line with animal welfare for fish and 3Rs [24, 25] are required.

Immunohistochemistry was employed as an additional method to demonstrate, *in situ*, the presence of *L*. *garvieae* in different organs in the present study. This method detects both viable bacteria at the time of sampling, and also unviable/inactivated bacteria including antigens in the vaccine used. It is therefore not surprising that *L*. *garvieae* was demonstrated in the vaccinated group where no corresponding bacteria was re-isolated., consistent with previous reports [26, 27]. The demonstration of bacteria by immunohistochemistry in the present study was more sensitive than bacterial re-isolation. This is in contrast with previous reports where the opposite was reported [28, 29], suggesting that the sensitivity of immunohistochemistry relative to pathogen re-isolation depends on several factors, including the type of pathogen in question. Nevertheless, apart from the vaccinated group where vaccine-associated inactivated antigens were present, estimation of bacterial loads in different tissues by immunohistochemistry and bacterial re-isolation were on average comparable.

In the present study, *L*. *garvieae* infection establishment in tilapia progressed very rapidly, peaking within 3–5 days. This is consistent with findings of others [18]. The distribution of *L*. *garvieae* in different tissues (based on the trends and frequency of infected organs over time) as observed from bacterial re-isolation (Table 3) and immunohistochemistry in the adjuvant and control groups in the present study (Table 4) suggests that bacterial localization following internationalization occurs firstly in the liver, kidney and spleen before spreading to the brain. These results are similar to the findings in other studies in rainbow trout following infection by different routes [30], although no chronological order of infection development was suggested in that study.

Nile tilapia immunised with oil-based *L*. *garvieae* vaccine in the present study produced antibodies significantly higher (p<0.001) than the controls or adjuvant only groups by 21 days post vaccination (Fig 4). This trend continued until 3 days post challenge when the antibody titres dropped sharply. The high antibody titres in the vaccinated group at the point of challenge and the absence of bacteria in tissues of this group as demonstrated by lack of bacterial re-isolation (Table 3) suggests that antibodies play a significant role in the protection of the fish against infection. This agrees with the mechanism of action of oil-adjuvanted vaccines and also known protective mechanisms against extracellular pathogens [31, 32]. The drop in antibody titres shortly after challenge is consistent with previous observations in Atlantic salmon [33] which reflected virus neutralization or the “consumption” of antibodies through antibody-antigen complex formation. Interestingly, the antibody titres in the vaccinated group in the present study increased from 5 dpc until the end of the experiment suggesting that following challenge, the bacteria acted as a boost. This was however not the case in the control or adjuvant only groups where no antibodies were detected.

## Conclusion

The results of the present study suggests that tilapia can be vaccinated and protected against L. garvieae by using inactivated oil adjuvanted autovaccines. However, because of the low challenge pressure used for challenge and the relatively small number per group of the fish, additional studies are required to confirm with greater certainty the findings of the present study. Bacteria were re-isolated from different organs and also demonstrated by immunohistochemistry while the antibody response was evaluated by elisa. This combination of findings provides an alterntive approach for testing vaccines that does not involve mortality. This is in line with fish welfare and the reduction in fish suffering (3Rs).

## Supporting information

S1 Data (XLSX)

S2 Data (XLSX)
